# The impact of the time of drug administration on the effectiveness of combined treatment of hypercholesterolemia with Rosuvastatin and Ezetimibe (RosEze): study protocol for a randomized controlled trial

**DOI:** 10.1186/s13063-017-2047-8

**Published:** 2017-07-11

**Authors:** Karolina Obońska, Michał Kasprzak, Joanna Sikora, Ewa Obońska, Krzysztof Racki, Natalia Goździkiewicz, Magdalena Krintus, Jacek Kubica

**Affiliations:** 1 Department of Cardiology and Internal Medicine, Nicolaus Copernicus University, Collegium Medicum, 9 Skłodowskiej-Curie Street, 85-094 Bydgoszcz, Poland; 2 Department of Pharmacology and Therapy, Nicolaus Copernicus University, Collegium Medicum, 9 Skłodowskiej-Curie Street, 85-094 Bydgoszcz, Poland; 3 Students Scientific Society, Department of Cardiology and Internal Medicine, Nicolaus Copernicus University, Collegium Medicum, 9 Skłodowskiej-Curie Street, 85-094 Bydgoszcz, Poland; 4 Department of Laboratory Medicine, Nicolaus Copernicus University, Collegium Medicum, 9 Skłodowskiej-Curie Street, 85-094 Bydgoszcz, Poland

**Keywords:** Adherence, Cardiovascular risk, Coronary artery disease, Dyslipidemia, Ezetimibe, Hydroxymethylglutaryl-CoA, Hypercholesterolemia, Morning/evening dosing, LDL-C, Rosuvastatin

## Abstract

**Background:**

Hypercholesterolemia is one of the main risk factors for cardiovascular disease. The first line treatment for hypercholesterolemia is statin therapy. When the expected low-density lipoprotein cholesterol (LDL-C) concentration is not achieved, the pharmacotherapy may be extended by combining the statin with the cholesterol absorption inhibitor ezetimibe.

**Methods/design:**

The study is designed as a randomized, open-label, single-center, crossover study evaluating the effectiveness of combined therapy with rosuvastatin and ezetimibe for hypercholesterolemia. The study is planned to include 200 patients with hypercholesterolemia ineffectively treated with statins for at least 6 weeks. After enrollment participants are randomized into one of two arms receiving rosuvastatin and ezetimibe. In the first arm the study drug is administered in the morning (8:00 am) for 6 weeks and then in the evening for the next 6 weeks; in the second arm the study drug is administered at first in the evening (8:00 pm) for the first 6 weeks and then in the morning for the following 6 weeks. In order to minimize non-adherence to the treatment, all patients will receive the study drug free of charge. The primary outcome of the study is change in LDL-C at 6 and 12 weeks of the treatment, depending on the time of day of study drug administration. The secondary endpoints include change in total cholesterol, high-density lipoprotein (HDL) cholesterol, triglycerides, apolipoproteins ApoB and Apo AI, non-HDL cholesterol, small, dense (sd)-LDL cholesterol, lipoprotein(a), glucose, glycated hemoglobin, high-sensitivity C-reactive protein, aspartate aminotransferase, alanine aminotransferase, gamma-glutamyl transferase, and creatine kinase at 6 and 12 weeks of the study drug treatment, as well as assessment of plasma fluorescence using stationary and time-resolved fluorescence spectroscopy at baseline and at 6 and 12 weeks of the therapy.

**Discussion:**

The RosEze trial is expected to demonstrate whether there is a significant difference in the effectiveness of the lipid-lowering therapy in reducing the concentration of cholesterol when the medications are taken in the morning compared with the evening time of day.

**Trial registration:**

ClinicalTrials.gov, NCT02772640. Registered on 28 March 2016.

**Electronic supplementary material:**

The online version of this article (doi:10.1186/s13063-017-2047-8) contains supplementary material, which is available to authorized users.

## Background

Hypercholesterolemia is one of the main risk factors for cardiovascular disease (CVD) [1]. Despite enormous progress in the treatment of coronary artery disease (CAD), patients after surviving their first episode are at risk of recurrence [2]. Hypercholesterolemia is a modifiable risk factor for CVD. Lifestyle changes [3], increased daily physical activity [4–7], as well as optimized diet [8–12] may lead to normalization of specific cholesterol fractions. This strategy, however, often fails or is not sufficient, thus providing the need for pharmacotherapy. The current guidelines recommend statins as the first choice drugs for the treatment of hypercholesterolemia up to the highest recommended dose or the highest tolerable dose (class of recommendation I, level of evidence A) [2]. According to a meta-analysis of studies assessing statins, each 1.0 mmol/L (~40 mg/dL) reduction in low-density lipoprotein cholesterol (LDL-C) corresponds to a 10% reduction in all-cause mortality and a 20% reduction in the number of deaths from CAD [13]. Furthermore, each 1 mmol/L (40 mg/dL) reduction in LDL-C translates into a 23% and 17% reduction of the risk of major coronary events and stroke, respectively. Similar results concerning the efficacy and safety of lipid-lowering therapy using statins were obtained in meta-analyses of studies on primary prevention [14–16]. Statins are a heterogeneous group of drugs with respect to their LDL-C reduction power. So far, the most potent statin is rosuvastatin. However, despite intensive statin therapy, only a small group of patients (approximately 20%) reach the therapeutic lipid-lowering goal [17–20]. When the LDL-C goal is not achieved, the combination of statin with a cholesterol absorption inhibitor — ezetimibe — may be considered (class of recommendation IIa, level of evidence B) [2]. Statin dose titration seems to be less effective compared with the combined therapy with statin and ezetimibe [21]. The combination of statin with ezetimibe reduces the LDL-C by an additional 15–20% [22].

Unfortunately, despite a wealth of evidence on the efficacy and effectiveness of statins in both primary and secondary prevention, statin adherence remains a consistent barrier, with rates below 50% demonstrated in several studies [2, 23]. Adherence declines over the duration of treatment [2, 24–28], and this phenomenon is even more pronounced in patients treated for primary compared with secondary prevention of CVD [2]. It was demonstrated in a systematic review and meta-analysis that poor adherence is not limited to statins but to all medications used in secondary prevention for CVD [2, 29, 30]. Furthermore, non-adherence translates into increased healthcare costs of morbidity, hospital readmissions, and mortality [31–35]. There are many determinants of non-adherence to different medications including statins [36–38]. One of the reasons for non-adherence is a large number of drugs taken daily by the patient. Thus, more benefit is achieved with combined drugs containing statin and ezetimibe. Tablets comprising both of these drugs (statin and ezetimibe) simplify the drug administration and increase the probability of drug compliance. Furthermore, the use of these tablets may translate into increased probability for achieving therapeutic goals in hypercholesterolemia treatment [39]. Taking into account the metabolism of cholesterol and possible drug-drug interactions, it is recommended to administer simvastatin in the evening [40]. Rosuvastatin can be administered at any time of the day [41]. In our everyday practice we meet many patients with hypercholesterolemia treated with statins. All of them take the statin in the evening; whereas, for combined treatment with ezetimibe, they take the latter in the morning.

Until now there were no studies assessing the effectiveness of the combined treatment of hypercholesterolemia with rosuvastatin and ezetimibe according to the timing of the drug administration. To fill this evidence gap, the goals of this study are to determine whether the time of the day of rosuvastatin and ezetimibe administration plays any role in the effectiveness of the drug, and to identify side effects of combined therapy with rosuvastatin and ezetimibe administered at the same time of the day. Furthermore, we aim to assess whether administration of lipid-lowering drugs in the morning improves adherence compared with their evening administration.

Considering the potency of rosuvastatin, which is further enhanced when administered in combination with ezetimibe, we expect significant reduction of LDL-C concentration. However, the role of the time of drug administration in this case is questionable and worth further evaluation.

## Methods

The study is designed as a randomized, open-label, single-center, crossover study evaluating the effectiveness of combined therapy with rosuvastatin and ezetimibe for hypercholesterolemia. The study is conducted with full respect to regulations established in the Declaration of Helsinki. The eligibility criteria for enrollment into the study include adult patients with hypercholesterolemia defined according to the European guidelines [2] and ineffectiveness of statin monotherapy in the treatment of hypercholesterolemia after at least 6 weeks. All study participants will have been on statin therapy due to secondary prevention indications. Furthermore, they are eligible for the study when, despite statin monotherapy, the LDL-C concentration is higher than 70 mg/dL. Key exclusion criteria include the following: active liver disease; unexplained persistent increase in serum transaminase levels, including more than three times the upper limit of normal activity of one of them; severe renal impairment (creatinine clearance <30 mL/min); myopathy; concomitant treatment with cyclosporine and gemfibrozil; pregnancy or lactation; women of childbearing age not using effective methods of contraception; symptoms of muscle damage after using statins or fibrates in the past; activity of creatine kinase of more than five times the upper limit of normal.

The study is provided by the Department of Cardiology, Antoni Jurasz University Hospital No. 1 in Bydgoszcz, Poland.

**Fig. 1:**
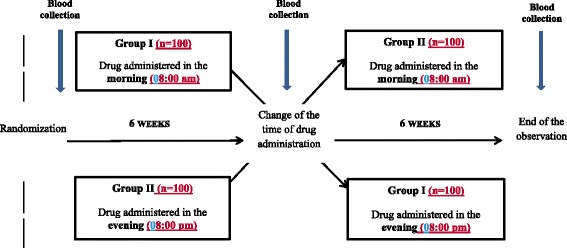
Scheme of the trial

**Fig. 2:**
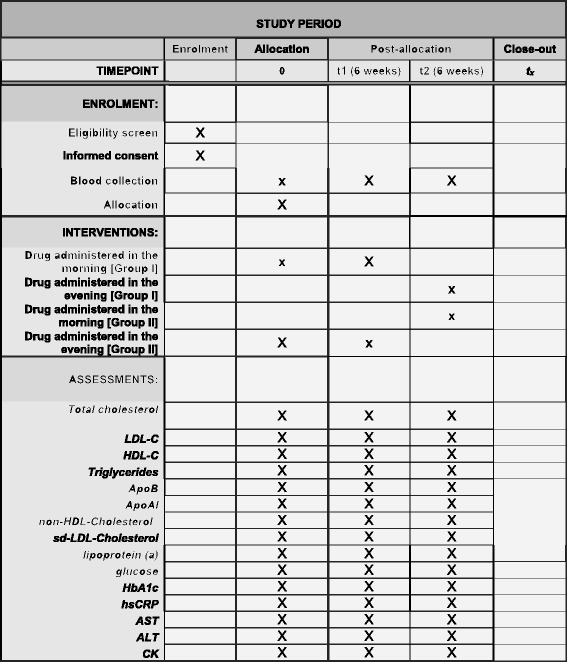
Detailed plan of the study (SPIRIT figure)

After the enrollment, all participants are randomized into one of two arms receiving rosuvastatin and ezetimibe. The study drug (rosuvastatin with ezetimibe) is given: (1) in the morning (8:00 am) for 6 weeks and then in the evening for the next 6 weeks in the first arm; (2) in the evening (8:00 pm) for the first 6 weeks and then in the morning for the following 6 weeks in the second arm. In order to encourage adherence to the treatment, all patients will receive the study drug free of charge over the entire observational period. We plan to enroll 200 patients with ineffectively treated hypercholesterolemia. The scheme of the study and detailed plan of the study are presented in Figs. 1 and 2, respectively. The Standard Protocol Items: Recommendations for Interventional Trials (SPIRIT) checklist is provided as Additional file 1.

### Endpoints

The primary outcome of the study is change in LDL cholesterol (LDL-C) at 6 (0 vs 6) and 12 (0 vs 12) weeks, as well as between the 6^th^ and 12th weeks, of study drug treatment (combination of ezetimibe and rosuvastatin), depending on the time of day of study drug administration.

The secondary endpoints include:

- Change in total cholesterol, high-density lipoprotein cholesterol (HDL-C), triglycerides (TGs), apolipoprotein B (ApoB), apoliprotein AI (Apo AI), non-HDL-cholesterol, small, dense-LDL-cholesterol (sd-LDL-cholesterol), lipoprotein(a) at 6 (0 vs 6) and 12 (0 vs 12) weeks, as well as between the 6th and 12th weeks of study drug treatment (combination of ezetimibe and rosuvastatin), depending on the time of day of study drug administration
- Assessment of glucose metabolism parameters: glucose, glycated hemoglobin (HbA1c) at baseline and at 6 (0 vs 6) and 12 (0 vs 12) weeks, as well as between the 6th and 12th weeks of treatment with study drug
- Assessment of high-sensitivity C-reactive protein (hsCRP) at baseline and at 6 (0 vs 6) and 12 (0 vs 12) weeks, as well as between the 6th and 12th weeks of treatment with study drug
- Assessment of aspartate aminotransferase (AST), alanine aminotransferase (ALT), gamma-glutamyl transferase (GGT), and creatine kinase (CK) at baseline and at 6 (0 vs 6) and 12 (0 vs 12) weeks, as well as between the 6th and 12th weeks of treatment with study drug
- Assessment of plasma fluorescence using stationary and time-resolved fluorescence spectroscopy at baseline, at 6 (0 vs 6) and 12 (0 vs 12) weeks, as well as between the 6th and 12th weeks of treatment with study drug

Apart from the analysis in the whole population, the above-mentioned endpoints will be analyzed in subgroups depending on age, sex, and presence of other comorbidities.

### Blood sample collection and laboratory measurements

Blood collection using an intravenous catheter (VACUTAINER, Becton Dickinson, Franklin Lakes, NJ, USA) is scheduled at the day of the enrollment and then during two follow-up visits, after 6 and 12 weeks. Laboratory tests are provided with the use of whole blood, serum, and plasma. Blood is collected in a fasting state, at least 12 h after the last meal, from the ulnar vein, in a volume of approximately 10 mL. Patients are also advised to abstain from alcohol and avoid excessive physical effort within 48 h preceding the blood collection. Serum tubes are allowed to clot for 30 min in a vertical position at room temperature. Serum is separated from venous blood samples by centrifugation for 10 min at 3000 × g at room temperature. Following the centrifugation, routine laboratory measurements are performed in fresh serum (glucose, creatinine, basic lipid profile [total cholesterol, LDL-C, HDL-C, TGs], AST, ALT, GGT, CK), and only HbA1c is measured in whole blood. All remaining serum is aliquoted and stored at —80 °C until assayed for hs-CRP, Apo AI, ApoB, lipoprotein(a), and sd-LDL-C. All measurements (except for HbA1c) are performed on the Horiba ABX Pentra 400 analyzer (Horiba ABX, Montpellier, France). LDL-C is measured directly and non-HDL-C is calculated. Reagents for lipoprotein(a) and sd-LDL-C (direct automated sdLDL-C kit) are supplied by Randox Laboratories (Crumlin, UK). HbA1c is measured on the BIO-RAD D-10™ Hemoglobin Testing System using high-performance liquid chromatography (HPLC).

Laboratory measurements are performed at the Department of Laboratory Medicine, Nicolaus Copernicus University, Collegium Medicum, Bydgoszcz, Poland, holding national and international procedures for quality control assays.

Blood samples for fluorescence measurements are collected at predefined time points. Plasma is separated from venous blood samples by centrifugation for 10 min at 3000 × g at room temperature. Plasma is filtered with the use of micro-dialyzers (Xpress Micro Dialyzer MD100, cut off 12–14 kDa) before fluorescence measurements. It is important to apply the preliminary fractionation to remove the majority of unnecessary particles from plasma just before the final measurement. In order to measure the fluorescence lifetime of samples, the stationary fluorescence spectrometer Hitachi F7000 and time-resolved spectrometer Life Spec II (Edinburgh Instruments Ltd) with the subnanosecond pulsed EPLED diode emitting a light of wavelength λ = 360 nm are used. The spectrometer Life Spec II is equipped with an electronically cooled photomultiplier Hamamatsu R928 connected with a TCC900 PC Card, which incorporates all the electronic modules required for time-correlated single photon counting (TCSPC). Additionally, the concentration of hydroxyproline in all samples is determined. Assessment of plasma fluorescence is provided at the Department of Pharmacology and Therapy, Nicolaus Copernicus University, Collegium Medicum.

### The statistical analysis

Since there is no reference study examining the effectiveness of combined treatment of hypercholesterolemia with rosuvastatin and ezetimibe according to timing of drug administration, we decided to perform an internal pilot study of 20 patients to estimate the final sample size. The means and standard deviations of reduction in LDL-C were 53.25 ± 31.49 mg/dL and 57.71 ± 30.35 mg/dL during morning and evening administration, respectively. The correlation coefficient between total cholesterol reduction during morning and evening drug administration was 0.901. Based on these results and assuming a two-sided alpha value of 0.005, we calculated using the *t* test for dependent variables that enrollment of 157 patients would provide a 98% power to demonstrate a significant difference in total cholesterol level. To compensate for potential withdrawal of consent or loss of study participants due to other reasons, we plan to enroll 200 patients.

The statistical analysis will be carried out using the Statistica 12.0 package (StatSoft, Tulsa, OK, USA). Normal distribution of quantitative variables will be assessed with the Shapiro-Wilk test. Depending on the results of the Shapiro-Wilk test parametric, the Student *t* tests, the one-way analysis of variance (ANOVA) or a non-parametric test (the Mann-Whitney *U* test, Wilcoxon’s signed rank test, or the Kruskal-Wallis ANOVA, multiple comparison test) will be used. The χ^2^ test, the χ^2^ with the Yates correction, or Fisher’s exact test will be used for qualitative variables depending on subgroup size. To assess factors influencing plasma fluorescence parameters, correlation analysis and multiple regression analysis will be conducted. Two-sided differences will be considered significant at *p* < 0.05.

### Safety of the trial

The study is limited only to patients with diagnosed hypercholesterolemia, in whom statin monotherapy does not allow one to reach the therapeutic goals. Moreover, all participants receive medications of all other groups recommended by the European Society of Cardiology guidelines accordingly to their comorbidities.

## Discussion

The RosEze trial is a phase IV, single-center, randomized, open-label, crossover study evaluating the effectiveness of combined therapy with rosuvastatin and ezetimibe for hypercholesterolemia depending on timing of the day of administration of the study treatment. This trial will reveal whether there is a significant difference in the effectiveness in reducing the concentration of cholesterol when the medications are taken in the morning compared with the evening time of the day. Considering that most medications are taken in the morning, it is possible that compliance with administration targets will improve if an effective dose can be taken in the morning instead of the evening.

### The study status

The study is currently recruiting participants. It was registered in the ClinicalTrials.gov database with identifier NCT02772640.

## Additional file

Additional file 1: SPIRIT checklist. (DOC 293 kb)

## Abbreviations

ALT: Alanine aminotransferase
Apo AI: Apolipoprotein AI
ApoB: Apolipoprotein B
AST: Aspartate aminotransferase
CAD: Coronary artery disease
CK: Creatine kinase
CVD: Cardiovascular disease
GGT: Gamma-glutamyl transferase
HbA1c: Hemoglobin A1c
HDL-C: High-density lipoprotein cholesterol
Hs-CRP: High-sensitivity C-reactive protein
LDL-C: Low-density lipoprotein cholesterol
