# Supplementary material for: The impact of the time of drug administration on the effectiveness of combined treatment of hypercholesterolemia with Rosuvastatin and Ezetimibe (RosEze): study protocol for a randomized controlled trial
Source: Trials. 2017 Jul 11;18:316. doi: 10.1186/s13063-017-2047-8 (PMC5504756; doi:10.1186/s13063-017-2047-8)
Supplement: Additional file 1: — SPIRIT checklist. (DOC 293 kb) [file 13063_2017_2047_MOESM1_ESM.doc]

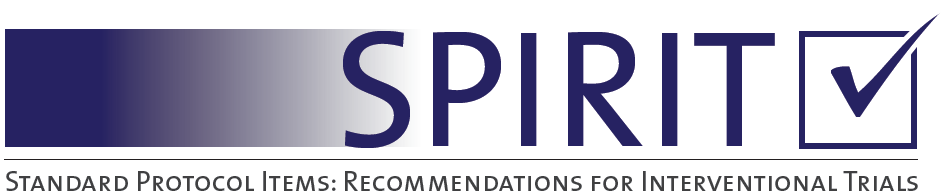


SPIRIT 2013 Checklist: Recommended items to address in a clinical trial protocol and related documents*

| Section/item | Item No | | Description | Addressed on page number |
| --- | --- | --- | --- | --- |
| **Administrative information** | | | |  |
| Title | 1 | Descriptive title identifying the study design, population, interventions, and, if applicable, trial acronym  The Impact of the Time of Drug Administration on the Effectiveness of Combined Treatment of Hypercholesterolemia with ROSuvastatin and EZEtimibe (RosEze): study protocol for a randomized controlled trial. | | ______1_______ |
| Trial registration | 2a | Trial identifier and registry name. If not yet registered, name of intended registry  ClinicalTrials.gov identifier: NCT02772640 | | _______4,13____ |
|  | 2b | All items from the World Health Organization Trial Registration Data Set | | Not applicable |
| Protocol version | 3 | Date and version identifier | | Not applicable |
| Funding | 4 | Sources and types of financial, material, and other support  The study is funded by Collegium Medicum of Nicolaus Copernicus University | | ______14_______ |
| Roles and responsibilities | 5a | Names, affiliations, and roles of protocol contributors  Karolina Obońska^1^, Michał Kasprzak^1^, Joanna Sikora^2^, Ewa Obońska^2^, Krzysztof Racki^1^, Natalia Goździkiewicz^3^, Magdalena Krintus^4^, Jacek Kubica^1^  1. Department of Cardiology and Internal Medicine, Nicolaus Copernicus University, Collegium Medicum, 9 Skłodowskiej-Curie Street, 85-094 Bydgoszcz, Poland  2. Department of Pharmacology and Therapy, Nicolaus Copernicus University, Collegium Medicum, 9 Skłodowskiej-Curie Street, 85-094 Bydgoszcz, Poland  3. Students Scientific Society, Department of Cardiology and Internal Medicine, Nicolaus Copernicus University, Collegium Medicum, 9 Skłodowskiej-Curie Street, 85-094 Bydgoszcz, Poland  4. Department of Laboratory Medicine, Nicolaus Copernicus University, Collegium Medicum, 9 Skłodowskiej-Curie Street, 85-094 Bydgoszcz, Poland.  KO conceived the study, participated in its design and its further development, and drafted the manuscript. JK participated in the design of this study and its further development and drafted the manuscript. MKa, JS, EO and MKr participated in the design of this study and its further development. KR and NG participated in the development of the study protocol | | _1  ____1___  14 |
|  | 5b | Name and contact information for the trial sponsor  Collegium Medicum, Nicolaus Copernicus University, 9 Skłodowskiej-Curie Street, 85-094 Bydgoszcz, Poland | | _______14______ |
|  | 5c | Role of study sponsor and funders, if any, in study design; collection, management, analysis, and interpretation of data; writing of the report; and the decision to submit the report for publication, including whether they will have ultimate authority over any of these activities | | Not applicable |
|  | 5d | Composition, roles, and responsibilities of the coordinating centre, steering committee, endpoint adjudication committee, data management team, and other individuals or groups overseeing the trial, if applicable (see Item 21a for data monitoring committee) | | Not applicable |
| Introduction |  |  | |  |
| Background and rationale | 6a | Description of research question and justification for undertaking the trial, including summary of relevant studies (published and unpublished) examining benefits and harms for each intervention  Hypercholesterolemia is one of the main risk factors for cardiovascular disease (CVD) [1]. Despite enormous progress in the treatment of coronary artery disease (CAD), patients after surviving their first episode are at risk of recurrences [2].Hypercholesterolemia is a modifiable risk factor for CVD-. Lifestyle changes [3], increased daily physical activity[4-7], as well as optimized diet [8-12] may lead to normalization of specific cholesterol fractions. This strategy, however, often fails or is not sufficientenough, thus providing the need forpharmacotherapy. The current guidelines recommend statins as the first choice drugs for the treatment of hypercholesterolemiaup to the highest recommended dose or the highest tolerable dose (class of recommendation I, level of evidence A) [2]. According to meta-analysis of studies assessing statins, each 1.0 mmol/L (~ 40 mg/dL) reduction in LDL-C corresponds to a 10% reduction in all-cause mortality and a 20% reduction in the number of deaths from CAD [13]. Furthermore, each 1 mmol/L (40 mg/dL) reduction in LDL-C translates into a 23% and 17% reduction of the risk of major coronary events and stroke, respectively. Similar results concerning the efficacy and safety of lipid-lowering therapy using statins were obtained in meta-analyzes of studies on primary prevention[14-16]. Statins are a heterogeneous group of drugs with respect to their LDL-C reduction power. So far, the most potent statin is rosuvastatin. Despite intensive statin therapy provided, only a small group of patients (approximately 20%)reach the therapeutic goal[17-20]. When the low-density lipoprotein cholesterol (LDL-C) goal is not achieved, the combination of statin with a cholesterol absorption inhibitor – ezetimibe may be considered (class of recommendation IIa, level of evidence B) [2].Statin dose titration seems to be less effective as compared with the combined therapy withstatin and ezetimibe [21]. The combination ofstatin with ezetimibe reduces the LDL-C by additional 15-20% [22].  Unfortunately, despite a wealth of evidence on the efficacy and effectiveness of statins in both primary and secondary prevention, statins adherence remains a consistent barrier, with rates below 50% demonstrated in several studies [2,23]. Adherence declines over the duration of treatment [2,24-28], and this phenomenon is even more pronounced in patients treated for primary compared with secondary prevention of CVD [2]. It was demonstrated in asystematic review and meta-analysis, that poor adherence is not limited to statins but to all medications used in secondary prevention for CVD [2,29,30].Furthermore, nonadherence translates into increased healthcare costs of morbidity, hospital readmissions and mortality [31-35]. There are many determinants of nonadherence to different medications including statins [36-38]. One of the reasons of nonadherence is a large number of drugs taken daily by the patient. Thus, more benefit is achieved with combined drugs containingstatin and ezetimibe. Tablets comprising both of these drugs (statin and ezetimibe) simplify the drug administration and increase probability of drug compliance. Furthermore, it may translate into increased probability for achieving therapeutic goals in hypercholesterolemia treatment [39].Taking into account the metabolism of cholesterol and possible drug-drug interactions it is recommended to administer simvastatin in the evening [40]. Rosuvastatin can be administered at any time of the day [41]. In our everyday practice we meet many patients with hypercholesterolemia treated with statins. All of them receive statin in the evening, whereas in case of combined treatment with ezetimibe they take the latter in the morning.  Up till now there were no studies provided assessing the effectiveness of combined treatment of hypercholesterolemia with rosuvastatin and ezetimibe according to timing of drug administration. To fill this evidence gap, the goal of this study is to determine whether time of the day of rosuvastatin and ezetimibe administration plays any role in the effectiveness of the drug, and to identify side effects of combined therapy with rosuvastatin and ezetimibe administered at the same time of the day. Furthermore, we aim to assess whether administration of lipid lowering drugs in the morning improves adherence compared with their evening administration.  Considering the potency of rosuvastatin, further enhanced when administered in combination with ezetimibe, we expect significant reduction of LDL cholesterol concentration. However the role of the time of drug administration in this case is questionable and worth further evaluation. | | 5-7 |
|  | 6b | Explanation for choice of comparators (as above) | | 7 |
| Objectives | 7 | Specific objectives or hypotheses  The aim of the study is to demonstrate, whether the time of day of administration of the study drug (containing rosuvastatin and ezetimibe) has an impact on the effectiveness of lipid-lowering therapy. | | 4 |
| Trial design | 8 | Description of trial design including type of trial (eg, parallel group, crossover, factorial, single group), allocation ratio, and framework (eg, superiority, equivalence, noninferiority, exploratory)  Phase IV, single center, randomized, open-label, cross-over study | | 3/7  _____________ |
| Methods: Participants, interventions, and outcomes | | | |  |
| Study setting | 9 | | Description of study settings (eg, community clinic, academic hospital) and list of countries where data will be collected. Reference to where list of study sites can be obtained  Department of Cardiology, Antoni Jurasz University Hospital no 1 in Bydgoszcz, Poland. | ______8_______ |
| Eligibility criteria | 10 | | Inclusion and exclusion criteria for participants. If applicable, eligibility criteria for study centres and individuals who will perform the interventions (eg, surgeons, psychotherapists)  The eligibility criteria for the enrollment into the study include adult patients with hypercholesterolemia defined according to the European guidelines [2], and ineffectiveness of statin monotherapy in the treatment of hypercholesterolemia after at least 6 weeks. All study participants were on statin therapy due to secondary prevention indications. Furthermore, they were found eligible for the study when despite statin monotherapy the LDL-C concentration was higher than 70 md/dl. Key exclusion criteria include: active liver disease; unexplained persistent increase in serum transaminase levels, including more than 3 times the upper limit of normal activity of one of them; severe renal impairment (creatinine clearance <30 mL/min); myopathy; concomitant treatment with cyclosporine, gemfibrozil; pregnancy and lactation; women of childbearing age not using effective methods of contraception; symptoms of muscle damage after using statins or fibrates in the past; the activity of creatine kinase>5 times the upper limit of normal. | ____7________ |
| Interventions | 11a | | Interventions for each group with sufficient detail to allow replication, including how and when they will be administered  After the enrollment all participants are randomized into one of two arms receiving rosuvastatin and ezetimibe. The study drug (rosuvastatin with ezetimibe) is given: 1) in the morning (8:00 am) for 6 weeks and then in the evening for the next 6 weeks in the first arm; 2) in the evening (8:00 pm) for the first 6 weeks and then in the morning for the following 6 weeks in the second arm. | ________8_____ |
|  | 11b | | Criteria for discontinuing or modifying allocated interventions for a given trial participant (eg, drug dose change in response to harms, participant request, or improving/worsening disease) | Not applicable |
|  | 11c | | Strategies to improve adherence to intervention protocols, and any procedures for monitoring adherence (eg, drug tablet return, laboratory tests)  In order to encourage adherence to the treatment, all patients will receive the study drug for free over the entire observational period. | 8 |
|  | 11d | | Relevant concomitant care and interventions that are permitted or prohibited during the trial | Not applicable |
| Outcomes | 12 | | The primary outcome of the study is change in LDL-cholesterol (LDL-C) at 6 (0 vs 6) and 12 (0 vs 12) weeks, as well as between the 6thand 12th week, of study drug treatment (combination of ezetimibe and rosuvastatin), depending on the time of day of study drug administration.  The secondary endpoints include:  - change in total cholesterol, high-density lipoprotein cholesterol (HDL-C), triglycerides (TG), apolipoprotein B (apoB), apoliprotein AI (APO AI), non–HDL-cholesterol, small dense-LDL-cholesterol (sd-LDL-cholesterol), lipoprotein (a) at 6 (0 vs 6) and 12 (0 vs 12) weeks, as well as between the 6th and 12th week, of study drug treatment (combination of ezetimibe and rosuvastatin), depending on the time of day of study drug administration;  - assessment of glucose metabolism parameters: glucose, glycated hemoglobin (HbA1c) at baseline and at 6 (0 vs 6) and 12 (0 vs 12) weeks, as well as between the 6th and 12th week of treatment with study drug;  - assessment of high sensitivity C - reactive protein (hsCRP) at baseline and at 6 (0 vs 6) and 12 (0 vs 12) weeks, as well as between the 6th and 12th weeks of treatment with study drug;  - assessment of aspartate aminotransferase (AST), alanine aminotransferase (ALT), gamma-glutamylotransferase (GGT) and creatine kinase (CK) at baseline and at 6 (0 vs 6) and 12 (0 vs 12) weeks, as well as between the 6th and 12th week of treatment with study drug;  - assessment of plasma fluorescence using stationary and time-resolved fluorescence spectroscopy at baseline, at 6 (0 vs 6) and 12 (0 vs 12) weeks, as well as between the 6th and 12th week, of treatment with study drug.  Apart from the analysis in the whole population above mentioned endpoints will be analyzed in subgroups depending on age, sex, and presence of other co-morbidities. | _____8-9_______ |
| Participant timeline | 13 | | Time schedule of enrolment, interventions (including any run-ins and washouts), assessments, and visits for participants. A schematic diagram is highly recommended (see Figure)  The study duration is 12 weeks. There are 3 visits planed: first at the time of enrolment, then after 6 weeks and the last one after next 6 weeks. Figure 1. | _____8________ |
| Sample size | 14 | | Estimated number of participants needed to achieve study objectives and how it was determined, including clinical and statistical assumptions supporting any sample size calculations  200 | ______8_______ |
| Recruitment | 15 | | Strategies for achieving adequate participant enrolment to reach target sample size | ______8_______ |
| **Methods: Assignment of interventions (for controlled trials)** | | | |  |
| Allocation: |  | |  |  |
| Sequence generation | 16a | | Method of generating the allocation sequence (eg, computer-generated random numbers), and list of any factors for stratification. To reduce predictability of a random sequence, details of any planned restriction (eg, blocking) should be provided in a separate document that is unavailable to those who enrol participants or assign interventions  Sealed envelopes. | 8 |
| Allocation concealment mechanism | 16b | | Mechanism of implementing the allocation sequence (eg, central telephone; sequentially numbered, opaque, sealed envelopes), describing any steps to conceal the sequence until interventions are assigned  Sealed envelopes arranged randomly. |  |
| Implementation | 16c | | Who will generate the allocation sequence, who will enrol participants, and who will assign participants to interventions  Authors. | _______1______ |
| Blinding (masking) | 17a | | Who will be blinded after assignment to interventions (eg, trial participants, care providers, outcome assessors, data analysts), and how | Not applicable______ |
|  | 17b | | If blinded, circumstances under which unblinding is permissible, and procedure for revealing a participant’s allocated intervention during the trial | Not applicable ____ |
| **Methods: Data collection, management, and analysis** | | | |  |
| Data collection methods | 18a | | Plans for assessment and collection of outcome, baseline, and other trial data, including any related processes to promote data quality (eg, duplicate measurements, training of assessors) and a description of study instruments (eg, questionnaires, laboratory tests) along with their reliability and validity, if known. Reference to where data collection forms can be found, if not in the protocol  Laboratory tests are provided at the Department of Laboratory Medicine, Nicolaus Copernicus University, Collegium Medicum, Bydgoszcz, Poland, holding national and international procedures for quality control assays. Assessment of plasma fluorescence is provided at the Department of Pharmacology and Therapy, Nicolaus Copernicus University, Collegium Medicum. Data will be collected at the Department of Cardiology, Antoni Jurasz University Hospital no 1 in Bydgoszcz, Poland. | ____10-11______ |
|  | 18b | | Plans to promote participant retention and complete follow-up, including list of any outcome data to be collected for participants who discontinue or deviate from intervention protocols | Not applicable |
| Data management | 19 | | Plans for data entry, coding, security, and storage, including any related processes to promote data quality (eg, double data entry; range checks for data values). Reference to where details of data management procedures can be found, if not in the protocol  Collected data will be stored in a database. It will be available at the Department of Cardiology, Antoni Jurasz University Hospital no 1 in Bydgoszcz, Poland. |  |
| Statistical methods | 20a | | Statistical methods for analysing primary and secondary outcomes. Reference to where other details of the statistical analysis plan can be found, if not in the protocol  Since there is no reference study examining the effectiveness of combined treatment of hypercholesterolemia with rosuvastatin and ezetimibe according to timing of drug administration, we decided to perform an internal pilot study of 20 patients to estimate the final sample size. The means and standard deviations of reduction in LDL-C was 53.25  ± 31.49 mg/dl and 57.71  ± 30.35 mg/dl during morning and evening administration respectively. The correlation coefficient between total cholesterol reduction during morning and evening drug administration was 0.901. Based on this results and assuming a two-sided alpha value of 0.005 we calculated using the t-test for dependent variables, that enrollment of 157 patients would provide an 98% power to demonstrate a significant difference in total cholesterol level. To compensate for potential withdrawal of consent or loss of study participants due to other reasons, we plan to enroll 200 patients.  The statistical analysis will be carried out using the Statistica 12.0 package (StatSoft, Tulsa, United States). Normal distribution of quantitative variables will be assessed with the Shapiro-Wilk test. Depending on results of the Shapiro-Wilk test parametric (the t-Student tests, the one-way ANOVA) or non-parametric test (the Mann-Whitney U-test, the Wilcoxon’s signed rank test, the Kruskal-Wallis ANOVA, multiple comparison test) will be used. The χ2 test, the χ2 with the Yates correction or the Fisher's exact test will be used for qualitative variables depending on subgroup size. To assess factors influencing plasma fluorescence parameters correlation analysis and multiple regression analysis will be conducted. Two-sided differences will be considered significant at p <0.05. | ____11_______ |
|  | 20b | | Methods for any additional analyses (eg, subgroup and adjusted analyses) | Not applicable |
|  | 20c | | Definition of analysis population relating to protocol non-adherence (eg, as randomised analysis), and any statistical methods to handle missing data (eg, multiple imputation) | - |
| **Methods: Monitoring** | | | |  |
| Data monitoring | 21a | | Composition of data monitoring committee (DMC); summary of its role and reporting structure; statement of whether it is independent from the sponsor and competing interests; and reference to where further details about its charter can be found, if not in the protocol. Alternatively, an explanation of why a DMC is not needed  Single center trial. | ______7_______ |
|  | 21b | | Description of any interim analyses and stopping guidelines, including who will have access to these interim results and make the final decision to terminate the trial | Not applicable |
| Harms | 22 | | Plans for collecting, assessing, reporting, and managing solicited and spontaneously reported adverse events and other unintended effects of trial interventions or trial conduct  All enrolled patients are given a telephone number to inform investigators in case of any adverse events. Then additional visit is scheduled. | ______-_______ |
| Auditing | 23 | | Frequency and procedures for auditing trial conduct, if any, and whether the process will be independent from investigators and the sponsor | Not applicable |
| Ethics and dissemination | | | |  |
| Research ethics approval | 24 | | Plans for seeking research ethics committee/institutional review board (REC/IRB) approval  The protocol of the study was approved by the Ethics Committee of Nicolaus Copernicus University in Toruń, Ludwik Rydygier Collegium Medicum in Bydgoszcz (approval number KB 618/2015). | _______13______ |
| Protocol amendments | 25 | | Plans for communicating important protocol modifications (eg, changes to eligibility criteria, outcomes, analyses) to relevant parties (eg, investigators, REC/IRBs, trial participants, trial registries, journals, regulators) | Not applicable |
| Consent or assent | 26a | | Who will obtain informed consent or assent from potential trial participants or authorised surrogates, and how (see Item 32)  Patients and investigators. | _______-______ |
|  | 26b | | Additional consent provisions for collection and use of participant data and biological specimens in ancillary studies, if applicable | Not applicable |
| Confidentiality | 27 | | How personal information about potential and enrolled participants will be collected, shared, and maintained in order to protect confidentiality before, during, and after the trial  Patients admitted to the Department of Cardiology, Antoni Jurasz University Hospital no 1 in Bydgoszcz will be screened for the eligibility to the study. The collected data will be held at the Department of Cardiology, Antoni Jurasz University Hospital no 1 in Bydgoszcz, Poland. | _______-______ |
| Declaration of interests | 28 | | Financial and other competing interests for principal investigators for the overall trial and each study site | Not applicable |
| Access to data | 29 | | Statement of who will have access to the final trial dataset, and disclosure of contractual agreements that limit such access for investigators  Only investigators. | - |
| Ancillary and post-trial care | 30 | | Provisions, if any, for ancillary and post-trial care, and for compensation to those who suffer harm from trial participation | Not applicable |
| Dissemination policy | 31a | | Plans for investigators and sponsor to communicate trial results to participants, healthcare professionals, the public, and other relevant groups (eg, via publication, reporting in results databases, or other data sharing arrangements), including any publication restrictions  Publications. | ______-_______ |
|  | 31b | | Authorship eligibility guidelines and any intended use of professional writers | Not applicable |
|  | 31c | | Plans, if any, for granting public access to the full protocol, participant-level dataset, and statistical code | Not applicable |
| Appendices |  | |  |  |
| Informed consent materials | 32 | | Model consent form and other related documentation given to participants and authorised surrogates | Attached below |
| Biological specimens | 33 | | Plans for collection, laboratory evaluation, and storage of biological specimens for genetic or molecular analysis in the current trial and for future use in ancillary studies, if applicable | Not applicable |

*It is strongly recommended that this checklist be read in conjunction with the SPIRIT 2013 Explanation & Elaboration for important clarification on the items. Amendments to the protocol should be tracked and dated. The SPIRIT checklist is copyrighted by the SPIRIT Group under the Creative Commons “[Attribution-NonCommercial-NoDerivs 3.0 Unported](http://www.creativecommons.org/licenses/by-nc-nd/3.0/)” license.

**Information about the study and consent form**

**Informacja dla uczestnika badania naukowego pt.: „Ocena skuteczności skojarzonego leczenia hipercholesterolemii rozuwastatyną i ezetymibem w zależności od pory dnia podawania leków”**

Rozpoznano u Pani/Pana hipercholesterolemię. Hipercholesterolemia jest to stan, w którym stężenia lipidów, zwłaszcza frakcji LDL-cholesterolu, nie odpowiadają wartościom uznanym za prawidłowe, a które wpływają na całkowite ryzyko sercowo-naczyniowe ocenione indywidualnie dla Pani/Pana. Hipercholesterolemia pozostaje jednym z głównych, modyfikowalnych czynników ryzyka rozwoju chorób sercowo-naczyniowych. Wystąpienie chorób sercowo-naczyniowych jest konsekwencją miażdżycy ścian tętnic i zakrzepicy, i jest główną przyczyną przedwczesnej śmiertelności oraz życia z niepełnosprawnością w Europie i w krajach rozwijających się. Głównymi postaciami klinicznymi chorób sercowo-naczyniowych są: choroba wieńcowa, niedokrwienny udar mózgu i choroba tętnic obwodowych. Oprócz podwyższonego stężenia cholesterolu, innymi modyfikowalnymi czynnikami ryzyka, które wpływają na rozwój CVD są: palenie tytoniu, brak aktywności fizycznej, nieprawidłowe nawyki żywieniowe, podwyższone ciśnienie tętnicze oraz cukrzyca typu 2. Do niemodyfikowalnych czynników ryzyka chorób sercowo-naczyniowych zaliczamy wiek i płeć męską. Normalizację nieprawidłowych wartości poszczególnych frakcji cholesterolu możemy osiągnąć za pomocą modyfikacji stylu życia poprzez optymalizację codziennej diety oraz wzrost aktywności fizycznej. Gdy jednak metody te nie są wystarczające należy zastosować odpowiednią farmakoterapię. Obecnie obowiązujące wytyczne Europejskiego Towarzystwa Kardiologicznego zalecają stosowanie statyn jako leków pierwszego wyboru w leczeniu hipercholesterolemii w dawce największej zalecanej lub największej tolerowanej celem osiągnięcia docelowych stężeń lipidów. W przypadku gdy nie osiągnięto docelowego stężenia cholesterolu frakcji LDL, można rozważyć skojarzenie statyny z inhibitorem wchłaniania cholesterolu (ezetymibem). W licznych badaniach wykazano, iż obniżenie wartości stężenia poszczególnych frakcji cholesterolu do wartości uznanych za prawidłowe powoduje zmniejszenie całkowitej śmiertelności oraz liczby zgonów z powodu choroby wieńcowej we wszystkich badanych grupach pacjentów zarówno w prewencji pierwotnej jak i wtórnej chorób sercowo-naczyniowych.

Proponujemy Pani/Panu udział w badaniu naukowym, którego celem jest ocena skuteczności skojarzonego leczenia rozuwastatyną i ezetymibem w leczeniu hipercholesterolemii w zależności od pory dnia podawania tych leków. W niniejszym badaniu terapia obniżająca stężenie frakcji LDL-cholesterolu złożona będzie z rozuwastatyny w dawce 20 mg oraz ezetymibu w dawce 10 mg. Leki te będą podawane Pani/Panu w postaci preparatu złożonego rano albo wieczorem wraz z innymi lekami, po losowym przypisaniu Pani/Pana do odpowiedniej grupy. Po upływie 6 tygodni nastąpi zmiana pory podawania leku – jeśli otrzymywała/otrzymywał Pani/Pan lek badany rano to będzie Pani/Pan otrzymywała/otrzymywał ten lek w godzinach wieczornych przez okres kolejnych 6 tygodni. Natomiast jeśli Pani/Pan otrzymywała/otrzymywał lek badany wieczorem to po zmianie będzie Pani/Pan otrzymywała/otrzymywał ten lek w godzinach porannych przez okres kolejnych 6 tygodni. Po upływie kolejnych 6 tygodni wszyscy uczestnicy badania będą otrzymywali rozuwastatynę i ezetymib w postaci oddzielnych preparatów: ezetymib w godzinach rannych i rozuwastatynę w godzinach wieczornych. W trakcie badania będą Państwo leczeni zgodnie z najnowszą wiedzą medyczną i obowiązującymi wytycznymi Europejskiego Towarzystwa Kardiologicznego. W dniu włączenia do badania zostanie pobrana od Pani/Pana próbka krwi żylnej o objętości 16 ml do badań laboratoryjnych (objętość porównywalna z objętością krwi pobieranej do rutynowych badań laboratoryjnych). Po 6 tygodniach od rozpoczęcia badania zostanie Pani/Pan zaproszona/y do Kliniki Kardiologii lub Przyklinicznej Poradni Kardiologicznej, przeprowadzona będzie wówczas wizyta kontrolna celem uzupełnienia obserwacji klinicznej oraz wykonania kontrolnych badań laboratoryjnych. Kolejne dwie wizyty kontrolne będą odbywały się również w odstępach 6 tygodniowych: po 6 tygodniach od zmiany pory podawania leku badanego (druga wizyta) oraz po 6 tygodniach przyjmowania oddzielnych preparatów: ezetymibu podawanego w godzinach rannych oraz rozuwastatyny podawanej w godzinach wieczornych (trzecia wizyta).

Wyniki przeprowadzonego badania, mogą w przyszłości posłużyć do skuteczniejszego leczenia pacjentów z hipercholesterolemią, obciążonych podwyższonym ryzykiem wystąpienia chorób sercowo-naczyniowych. Udział w badaniu wiąże się z niewielkim ryzykiem wystąpienia objawów ubocznych stosowanych leków oraz niewielkim ryzykiem ewentualnych powikłań miejscowych podczas pobierania krwi do badań labolatoryjnych.

Udział w badaniu jest dobrowolny i nieodpłatny. W każdej chwili istnieje możliwość odmowy i rezygnacji z uczestnictwa w badaniu bez konieczności podania przyczyny i bez jakichkolwiek konsekwencji czy zmiany sposobu leczenia.

Zobowiązujemy się do zachowania poufności tej części dokumentacji medycznej, która pozwoliłaby na identyfikację osoby uczestniczącej w badaniu oraz wyłączenia danych osobowych z ewentualnych publikacji wyników badania.

Miejscowość, data

Podpis pacjenta

**Formularz świadomej zgody na udział w badaniu naukowym pt.: „Ocena skuteczności skojarzonego leczenia hipercholesterolemii rozuwastatyną i ezetymibem w zależności od pory dnia podawania leków”**

*Zapoznałem się / zapoznałam się z treścią informacji o badaniu.*

*Wyrażam świadomą dobrowolną zgodę na udział w w/w eksperymencie badawczym.*

*Zostałem/am poinformowany/a o możliwości zadawania pytań prowadzącemu badanie*

*i otrzymania odpowiedzi na te pytania oraz o możliwości odstąpienia od udziału w badaniu*

*w każdym jego stadium.*

*Wyrażam zgodę na przetwarzanie przez osobę lub podmiot przeprowadzający badanie danych związanych z udziałem w badaniu.*

Imię i nazwisko pacjenta

Adres pacjenta

Nr historii choroby

Miejscowość, data

Podpis pacjenta
